# Magnitude and determinants of undernutrition among tuberculosis patients in Ethiopia: systematic review and meta-analysis

**DOI:** 10.1186/s12889-024-19220-3

**Published:** 2024-06-25

**Authors:** Jira Wakoya Feyisa, Robera Demissie Berhanu, Matiyos Lema, Markos Desalegn, Emiru Merdassa, Keno Melkamu Kitila, Wase Benti Hailu, Sidie Debelo Beyena, Adisu Tafari Shama

**Affiliations:** 1 Department of Public Health, Institute of Health Sciences, Wollega University, P.O.BOX: 395, Nekemte, Ethiopia; 2 School of Nursing and Midwifery, Institute of Health Sciences, Wollega University, Nekemte, Ethiopia; 3 Department of Public Health, College of Health Sciences, Mettu University, Mettu, Ethiopia

**Keywords:** TB, Undernutrition, Meta-analysis, Ethiopia

## Abstract

**Background:**

Undernutrition increases the risk of TB infection to be active TB, death and relapse of the disease. Undernutrition also disturbs the management process of tuberculosis. Therefore, this study aimed to estimate the pooled magnitude and determinants of undernutrition among TB patients in Ethiopia.

**Methods:**

From August 20, 2022 to January 6, 2023, the research articles were identified via the search engines Google Scholar, Medline, Pub Med, Cochrane Library, and Web of Science. Stata version 14 was used for analysis, along with a standardized data extraction checklist. The Cochrane Q test statistic and I2 statistics were used to determine heterogeneity. A random-effect model was used to assess the extent of undernutrition among TB patients. OR with a 95% CI was used to report the relationship between undernutrition and independent factors. A funnel plot and Egger’s test were used to examine publication bias.

**Results:**

A total of 720 research articles were identified via several databases and 21 studies were included in the systematic review and meta-analysis. The pooled magnitude of undernutrition among TB patients was 48.23% (95% CI 42.84, 53.62). The current meta-analysis revealed that patients who had no formal education (OR = 2.11(95%CI: 1.09, 4.06), average monthly income < 1800 ETB (OR = 2.32 (95CI: 1.33, 4.04), unable to work (OR = 2.61(95CI:1.99, 3.43), patients who had eating disorder (OR = 2.73 (95CI: 2.09, 3.56), patients who had intestinal parasite (OR = 3.77 (95CI: 2.39, 5.94), patients of > 5 family size (OR = 3.79 (95CI: 1.06, 14.93), and patients who drank alcohol (OR = 1.47(95CI: 1.06, 2.05) were significantly associated with undernutrition.

**Conclusion:**

This meta-analysis examined the high magnitude of undernutrition among TB patients in Ethiopia. Strategic and police-oriented intervention to prevent factors contributing to the problem is mandatory.

## Introduction

Tuberculosis (TB) is an immune-compromising disease caused by Mycobacterium Tuberculosis complex and other related species and usually affects the lungs but almost all organs can be affected [1]. Thus it is conveniently classified into; Pulmonary TB (PTB) and Extra-pulmonary TB (EPTB) [1]. Early diagnosis of infectious TB cases and treating them effectively are the keystones of global TB control programs [2]. Despite the exhaustive strategies of the World Health Organization for controlling this disease, millions of people are still being infected annually [3].

Globally, TB is a common cause of mortality and morbidity which is estimated to be 10.6 million TB patients in 2021 increasing by 4.5% from 10.1 million in 2020 whereas, the estimated number of deaths from TB was 1.6 million which is common in developing countries [4]. Undernutrition increases the risk of TB infection to active TB, death, and relapse of the disease [5]. Undernutrition also disturbs the management process of tuberculosis [6]. Poor feeding and dietary practices inhibit the fight against TB especially in low-income countries as well as the body of a person suffering from TB has an increased request for calories, which often leads a TB patient to significant weight loss and this can aggravate acute undernutrition [7]. TB patients who are malnourished have more severe diseases, which increases the chance of mortality and severe acute undernutrition [8]. Evidence put forward that undernutrition among TB patients became associated factors for two- to four-fold times more likely in increasing mortality and a five-fold risk of drug-induced hepatotoxicity [9].

Identification of the pooled magnitude and determinants of undernutrition among TB patients in Ethiopia could have a great role for appropriate intervention to track the problem, which is a very common problem throughout the country. Thus, this systematic review and meta-analysis helps to have pooled evidence for the implementation strategy and to get policy attention.

## Methods

### Search strategy

From the very beginning, the PROSPERO database and the database of abstracts of reviews of effects (DARE) (http://www.library.UCSF.edu) were searched to see whether there were any published or ongoing projects related to the topic. This systematic review and meta-analysis study protocol has been submitted to the International Prospective Register of Systematic Reviews (PROSPERO) and has been assigned the registration ID, which is CRD42023422305. The research articles search strategy, selection of studies, data extraction, and result reporting were done in accordance with Preferred Reporting Items for Systematic Reviews and Meta-Analyses (PRISMA) guidelines [10, 11]. A PICO principle was adapted for searching terms. The research articles used for this systematic review and meta-analysis were identified through Google Scholar, Medline/Pub Med, Cochrane Library, the Web of Science, Hinari, Science Direct, ProQuest, African Journals Online, and online university repositories (University of Gondar, Addis Ababa, Jimma, and Haramaya University) search engines by developing search strategies. Boolean operators such as OR, AND, and NOT were used with search terms such as prevalence”, “magnitude”, “proportion”, “burden”, “undernutrition”, “malnutrition”, “malnourishment”, “underweight”, “tuberculosis”, “factors”, “determinants”, “Predictors”, “adults”, “Ethiopia. Identified research articles were screened to make sure that all relevant literature was included. Literature was downloaded to Endnote (version X7.8) to maintain and manage citations, and facilitate the review process [12].

### Eligibility criteria

In this systematic review and meta-analysis, we included all studies that were conducted on undernutrition among adult TB patients and/or associated factors in Ethiopia. The participants were Adult TB patients. We included all types of articles that were published in the form of journal articles, master theses, and dissertations in the English language. Full research articles, which were not accessed after at least two email contacts of the primary author, were not included because of the failure to assess the quality of articles in the absence of full text. All studies conducted in Ethiopia were included.

### Outcome measurement

There were two main outcomes. The first outcome of interest was the magnitude of undernutrition (BMI < 18.5 kg/m2) among adult tuberculosis patients which was determined by dividing the number of patients having undernutrition by the total number of study subjects included in the final analysis. From the primary studies, undernutrition was operationalized as a BMI < 18.5 kg/m2. The second outcome was the factors associated with undernutrition, which was determined using the odds ratio (OR) and calculated based on binary outcomes from the included primary studies.

### Data collection and quality assessment

To assess the quality of the included study, the Joanna Briggs Institute (JBI) quality appraisal checklist for Observational studies quality assessment tool was used [13]. Five data extractors (JW, RD, AT, MD, and ML) used a Microsoft Excel standardized data extraction checklist for data extraction. Reference management software (Endnote version X7.8) was used to combine search results from databases and to remove duplicate articles initially. Then, research articles were screened and excluded by their abstracts and titles. Full-text articles or reports were assessed for the remaining research articles. Based on the preset inclusion and exclusion criteria, the eligibility of the primary studies was evaluated. For the first outcome (magnitude of undernutrition among TB patients), the data extraction checklist included the authors’ name, Year of publication, region (an area where studies were conducted), study design, sample size, response rate, and the number of participants with undernutrition. For the second outcome (factors associated with undernutrition among TB patients), data were extracted in two-by-two-tables format, and then the log OR was computed based on the findings of the primary articles. Inconsistencies between independent reviewers were fixed by including other reviewers (EM, and KM) after discussion for possible agreement. Corresponding authors of the research articles were contacted via email when the included primary articles did not have sufficient data.

### Data analysis and synthesis

The necessary information from each original study was retrieved using a format created in a Microsoft Excel spreadsheet. The data were then exported to STATA version 14.0 and used to determine the pooled effect size with 95% confidence interval. The Cochran Q test (Chi-squared statistic) and I2 statistic on forest plots were computed to assess heterogeneity among the included studies. At p 0.05, Cochran’s Q statistical heterogeneity test is declared statistically significant. I2 statistics range from 0 to 100%, with I2 statistic values of 0, 25, 50, and 75% indicating no, low, moderate, and high degrees of heterogeneity [14, 15]. When there was a high degree of heterogeneity for the first and second outcomes, a random-effects model was used to determine the pooled magnitude of undernutrition among TB patients and the pooled effect size of determinants. To determine the source of potential random variation, subgroup analysis was performed depending on the region of the studies and the type of study design. Meta-regression was used to determine the presence of statistically significant heterogeneity based on sample size and year of publication. A funnel plot was used to evaluate publication bias.

**Fig. 1:**
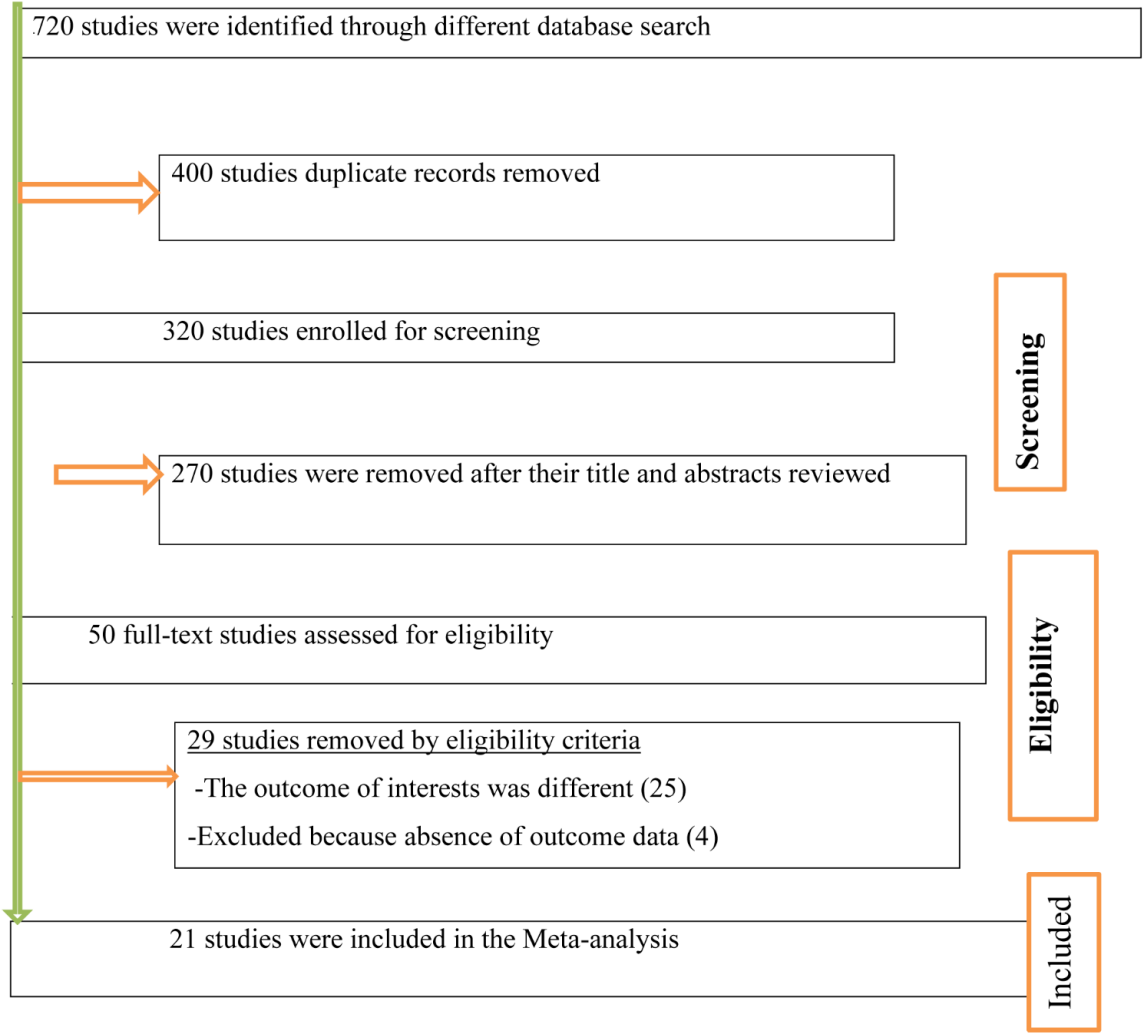
PRISMA flow diagram of included studies in the systematic review and meta-analysis of the magnitude and determinants of undernutrition among tuberculosis patients in Ethiopia, 2023

A total of 720 studies were identified from various electronic databases and library catalogs. Of these studies, 400 articles records were identified and removed due to duplication. Reviewing titles and abstracts resulted in the exclusion of 270 irrelevant research articles for our study. After assessing the full texts of the remaining articles, 29 studies were excluded as they did not meet the preset eligibility criteria. The remaining twenty-one (21) studies were included in the final systematic review and meta-analysis (Fig. 1).

### Characteristics of included studies

All of the twenty-two studies included in this study were published from 2006 to 2023 in peer review journals [16–36]. A total of 8001 study participants were included in the current systematic review and meta-analysis. The smallest sample size was 95 from a study conducted in the Dire Dawa [27], and the largest sample size was 1681 from a study conducted in the Amhara region [29]. Fifteen included studies were cross-sectional in study design [16–21, 24–26, 28–32, 36], three were retrospective cohort studies [22, 23, 34], and three were case-controls [27, 33, 35]. Regarding study setting, six studies were conducted in the Amhara region [17, 25, 28, 29, 34, 36] two studies in the SNNPR [22, 30], one study was from the Somali region [18], six studies were conducted in the Oromia region [16, 18, 20, 26, 31, 35], two studies were conducted in Tigray [21, 34], two studies were conducted in Addis Ababa [23, 32] and two studies were from Dire Dawa [24, 27]. The summary of the included articles is described in (Table 1).

**Table 1:** Summary of included studies in the systematic review and meta-analysis of the magnitude and determinants of undernutrition among tuberculosis patients in Ethiopia, 2023

SN	Author	Publication year	region	Area	Study design	sample size	quality score	Prevalence with 95%CI
1	Tadesse et al. [16]	2023	Oromia	Haramaya	Cross-sectional	330	9	43.64(38.29, 48.99)
2	Endalkachew et al. [17]	2022	Amhara	referral hospitals	Cross-sectional	405	8	42.22 (37.41, 47.03)
3	Brhane et al. [21]	2021	Tigray	central and northwest Zone	Cross-sectional	406	8	45.57(40.72, 50.41)
4	Tesfaye et al. [18]	2021	Oromia	Shashamene	Cross-sectional	368	9	29.08(24.44, 33.72)
5	Muse et al. [19]	2021	Somali	Jijiga	Cross-sectional	296	7	44.26(38.60, 49.91)
6	Hussien et al. [20]	2021	Oromia	9 zones in Oromia	Cross-sectional	450	8	51.56(46.94, 56.17)
7	Bade et al. [22]	2021	SNNPR	Southwest	Cohort	200	8	27.50(21.31, 33.69)
8	Seid et al. [23]	2020	Addis Ababa	Addis Ababa	Cohort	284	8	46.48(40.68, 52.28)
9	Abate et al. [25]	2020	Amhara	Bahir dar	Cross-sectional	368	7	43.48(38.41, 48.54)
10	Admassu et al. [25]	2020	DireDawa	Dire Dawa and Harari	Cross-sectional	452	8	50.22(45.6, 54.83)
11	Feleke et al. [29]	2019	Amhara	region	Cross-sectional	1681	9	57.16(54.80, 59.53)
12	Hussien et al. [26]	2019	Oromia	Bale	Cross-sectional	372	9	63.17(58.27, 68.07)
13	Gashaw et al. [28]	2019	Amhara	Wollo Zone	Cross-sectional	384	8	50.00(45.00, 55.00)
14	Hassen et al. [27]	2019	DireDawa	DireDawa	case-control	95	7	83.16(75.63, 90.68)
15	Geberemeskel et al. [30]	2018	SNNPR	Hosana	Cross-sectional	247	7	38.87 (32.78, 44.95)
16	Guadie, F et al. [31]	2016	Oromia	Adama	Cross-sectional	285	9	52.63(46.84, 58.43)
17	Dargie et al. [32]	2016	Addis Ababa	Addis Ababa	Cross-sectional	360	8	26.67(22.10, 31.24)
18	Ephrem et al. [33]	2015	Oromia	Ambo	case-control	104	7	63.46(54.21,72.72)
19	Wassie et al. [34]	2014	Amhara	Gondor	Cohort	384	8	32.813 28.116 37.509
20	Fisseha et al. [35]	2014	Tigray	Tigray region	case-control	230	9	56.09(49.67, 62.50)
21	kassu et al. [36]	2006	Amhara	Gondor	Cross-sectional	155	7	68.39 (61.07, 75.71)

### Magnitude of undernutrition among tuberculosis patients in Ethiopia

**Fig. 2:**
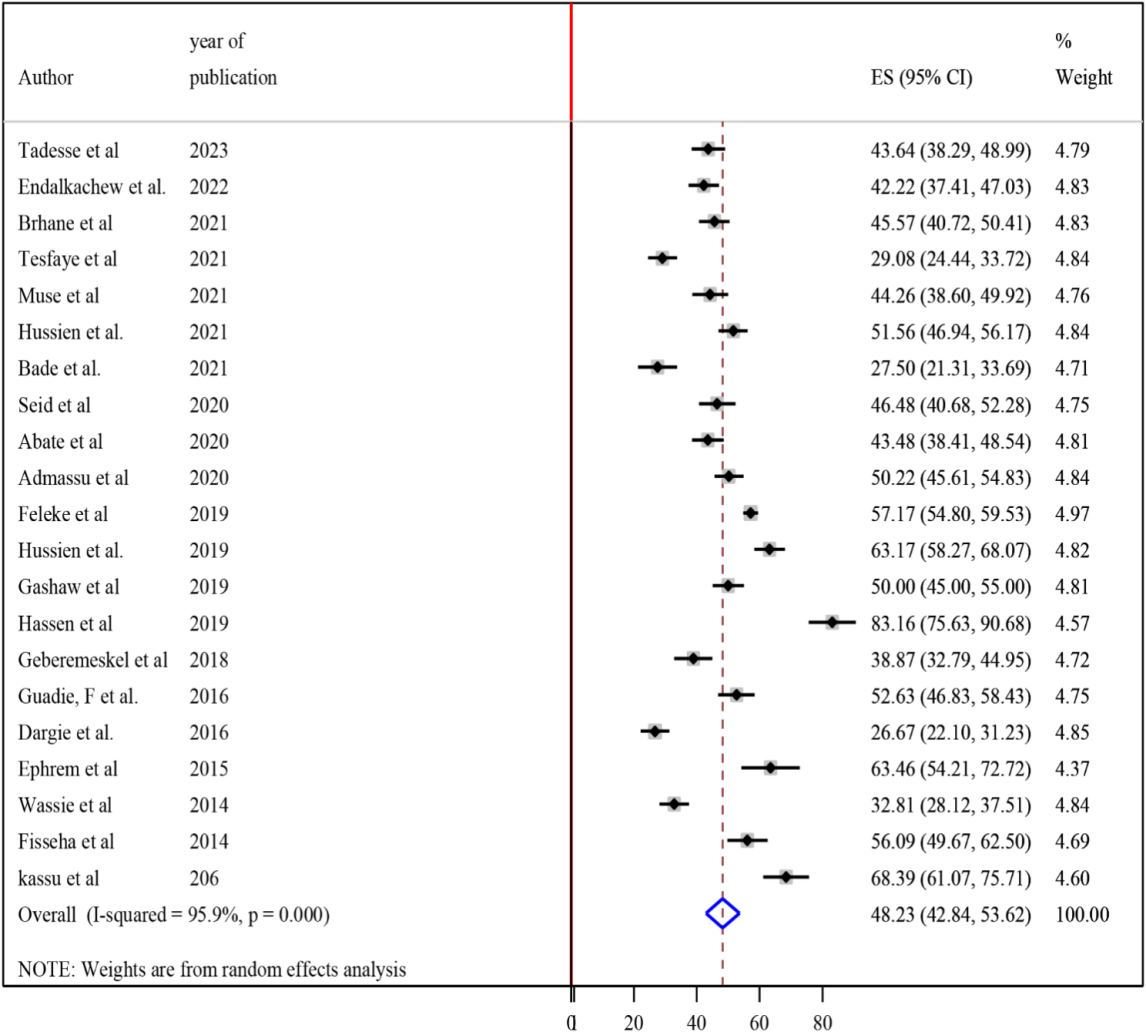
Forest plot of the pooled estimate of the magnitude of undernutrition among tuberculosis patients in Ethiopia, 2023

High heterogeneity was observed across the included studies (I2 = 95.9, *p* < 0.001) thus, a random-effects model was used to estimate the pooled magnitude of undernutrition among tuberculosis patients in Ethiopia. The pooled magnitude of undernutrition among tuberculosis patients was 48.23% (95% CI 42.84, 53.62). The highest 83.16% (95% CI 75.63, 90.68) magnitude was observed in a study conducted in Dire Dawa [27] and the lowest 26.67% (95% CI 22.1 31.24) magnitude was observed in a study conducted in Addis Ababa [32] (Fig. 2).

To check for underlying heterogeneity, meta-regression models were done by using sample size and year of publication, but there was statistically insignificant underlying heterogeneity (*p* = 0.861) and (*p* = 0.158), respectively (Table 2).

**Table 2:** Meta-regression analysis based on sample size and year of publication

Variables	Coefficients	*p*-value
Year of publication	− 0.0118954	0.158
Sample size	0.0017464	0.861

### Subgroup analysis

**Fig. 3:**
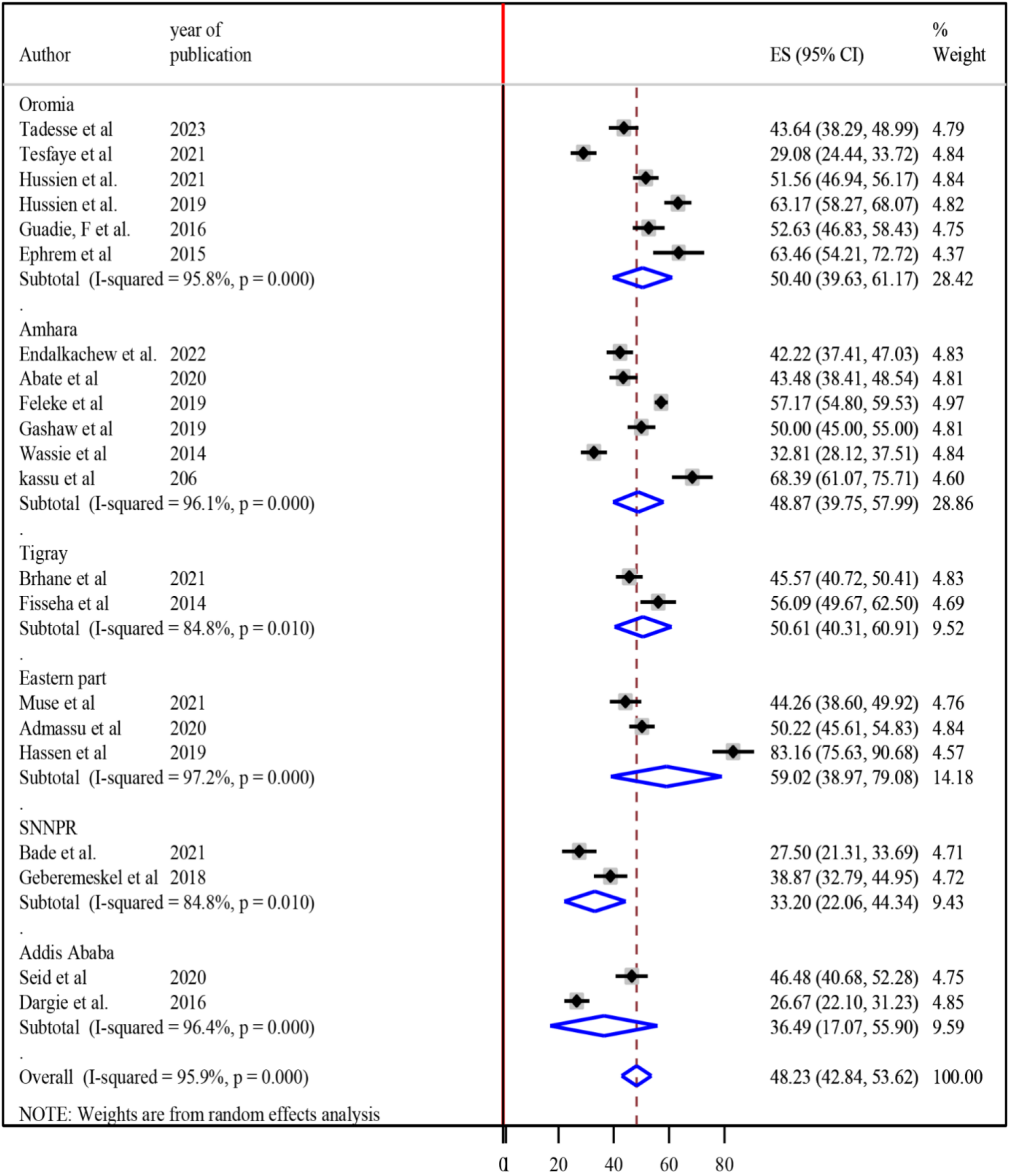
Subgroup analysis of the magnitude of undernutrition among tuberculosis patients in Ethiopia based on the region, 2023

**Fig. 4:**
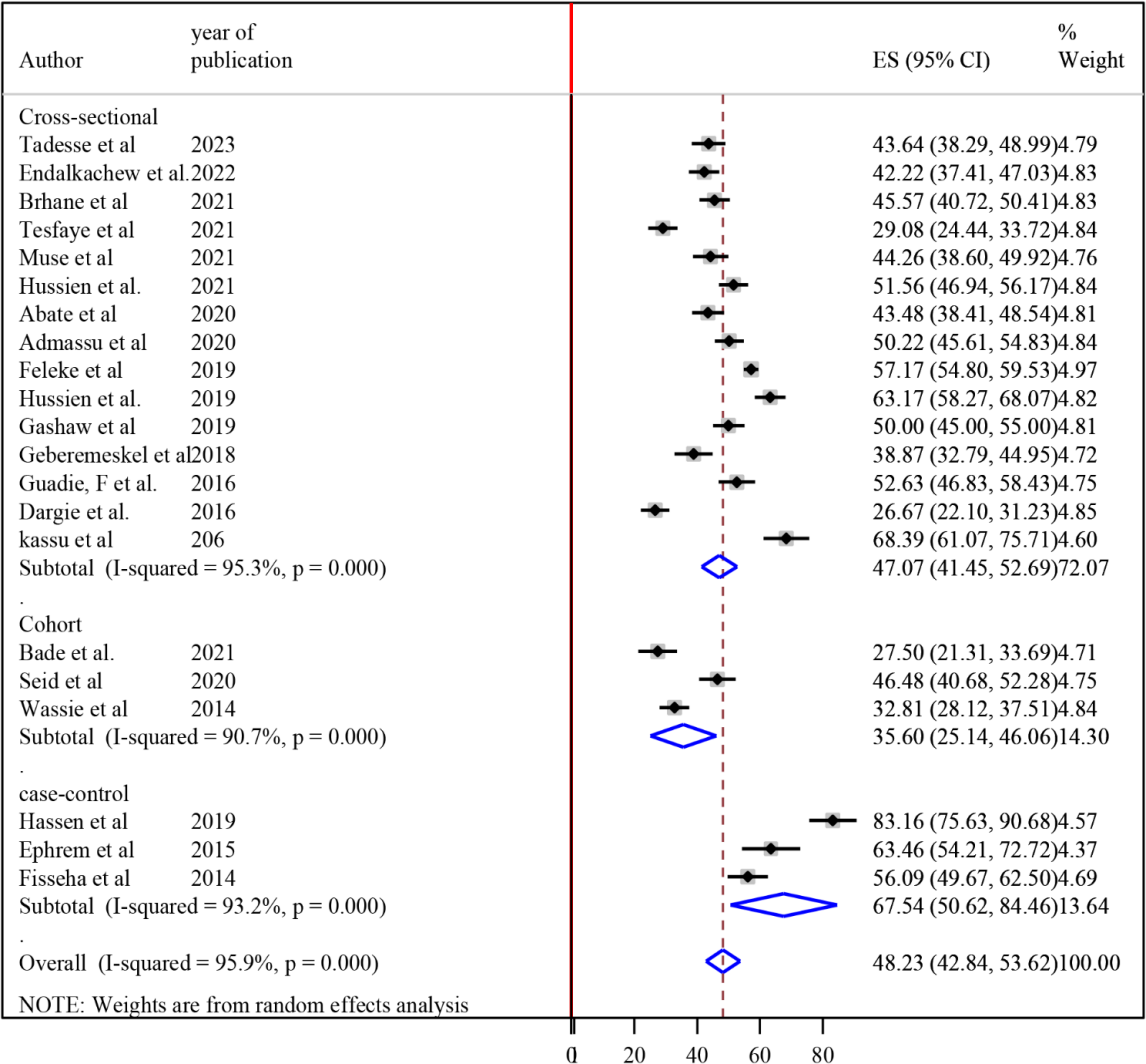
Subgroup analysis of the magnitude of undernutrition among tuberculosis patients in Ethiopia based on the study design, 2023

To realize heterogeneity among the included articles, subgroup analysis was undertaken by study setting and study design. According to where the studies conducted, the highest magnitude of undernutrition among tuberculosis patients 59.02% (95% CI 38.97, 79.08) was observed in the eastern part (Dire Dawa, Harari and Somali) [19, 24, 27] and the lowest magnitude of undernutrition among tuberculosis patients was 33.20% (95% CI 22.06, 44.34) was reported in SNNPR [22, 30] (Fig. 3). According to the study design of the studies, the magnitude of undernutrition among tuberculosis patients among cross-sectional, case-control and cohort studies were 47.07% (95% CI 41.45, 52.69), 67.54% (95% CI 50.62, 84.47) and 35.60% (95%CI 25.14, 46.07) respectively (Fig. 4).

### Publication bias

**Fig. 5:**
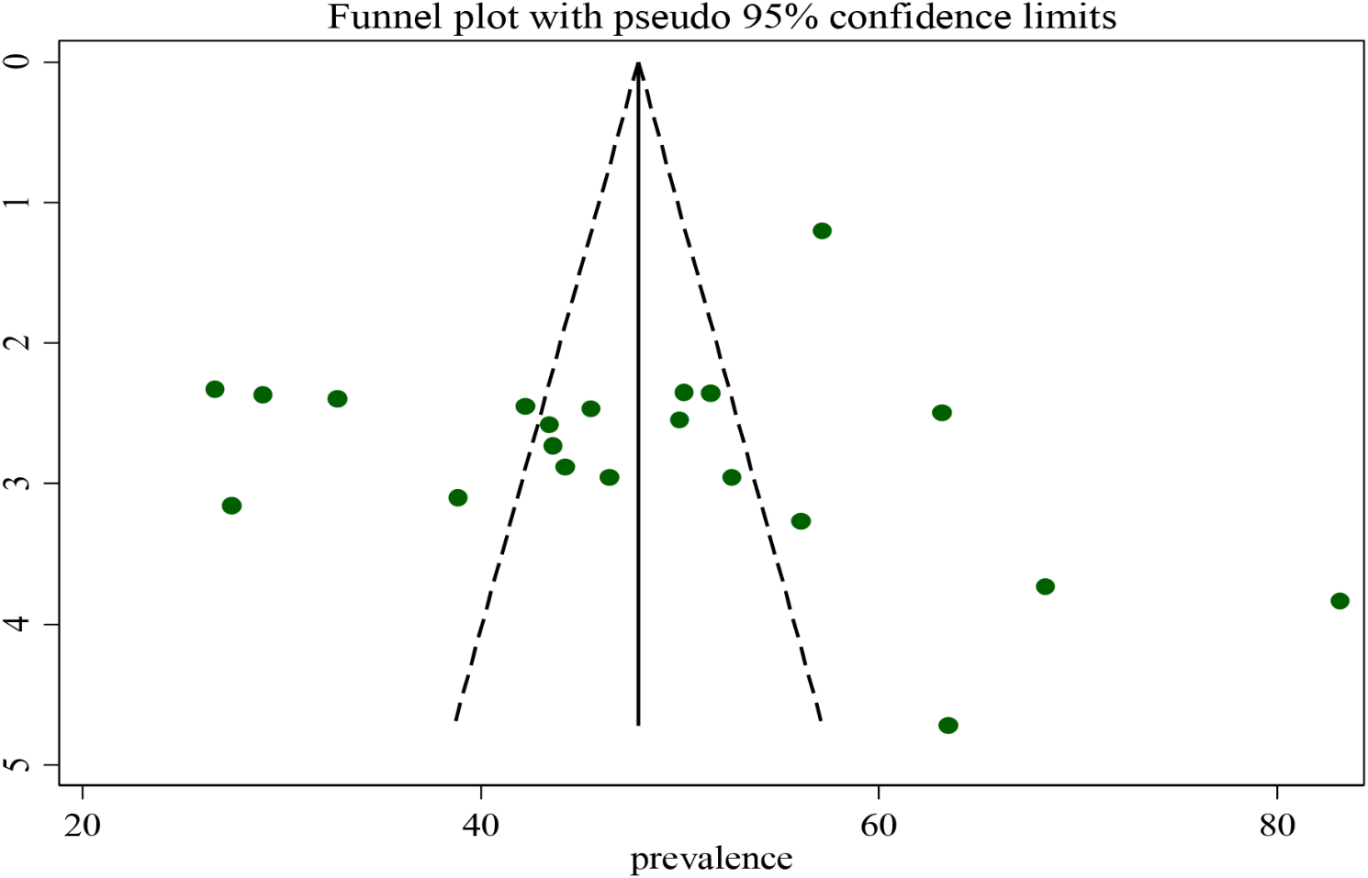
Funnel plot with 95% confidence limit of the magnitude of undernutrition among tuberculosis patients in Ethiopia, 2023

To test the existence of publication bias, the graphical funnel plot and Egger’s test at a 5% significance level were executed. The visual examination of the funnel plot presented symmetrically which is an indicator for the absence of publication bias (Fig. 5). Egger’s and begg test also showed the absence of publication bias at a 5% significance level at p- value (0.785) and (0.131) respectively.

### Sensitivity analysis

**Fig. 6:**
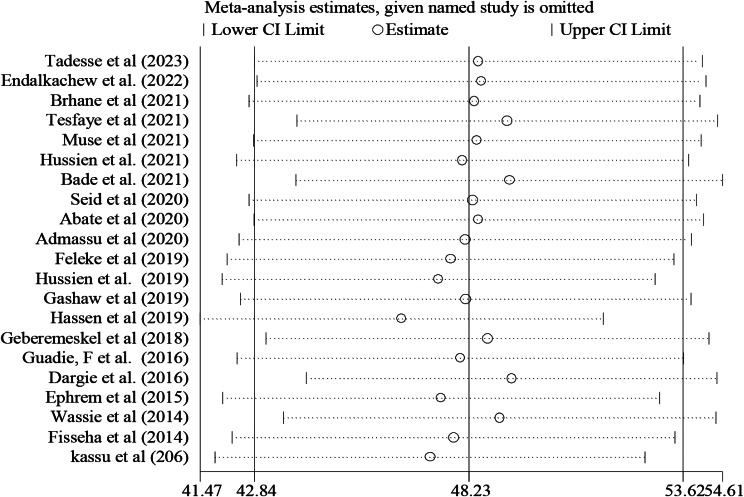
Result of sensitivity analysis of the magnitude of undernutrition among tuberculosis patients in Ethiopia, 2023

Sensitivity analysis was done to see any outliers and it showed there was no single study influence on the overall included studies (Fig. 6).

### Factors associated with undernutrition among tuberculosis patients

#### Association between Education and undernutrition among tuberculosis patients

**Fig. 7:**
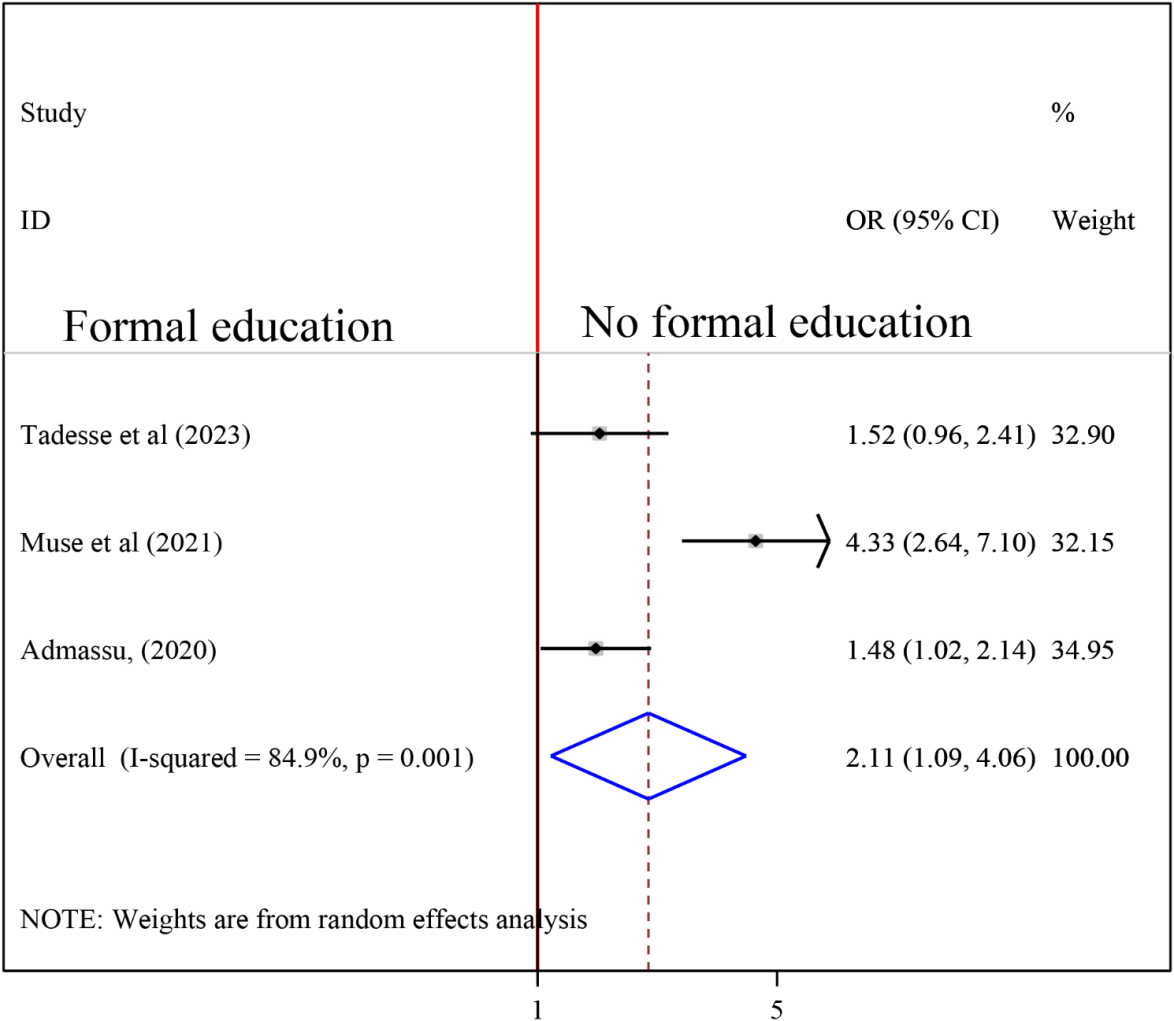
Forest plot of the pooled estimate of the association between education and undernutrition among tuberculosis patients in Ethiopia, 2023

To investigate the association between educational status and undernutrition among tuberculosis patients, three studies were included in the analysis [16, 19, 24]. A random-effects model was utilized to evaluate the association between education and undernutrition among tuberculosis patients (I2 = 84.9%, P-value = 0.001). The result of the analysis showed that the association between education and undernutrition among tuberculosis patients was statistically significant (OR = 2.11(95%CI: 1.09, 4.06). TB patients those who had no formal education were 2.11 times more likely to develop undernutrition than who had formal education (Fig. 7).

### Association between age and undernutrition among tuberculosis patients

**Fig. 8:**
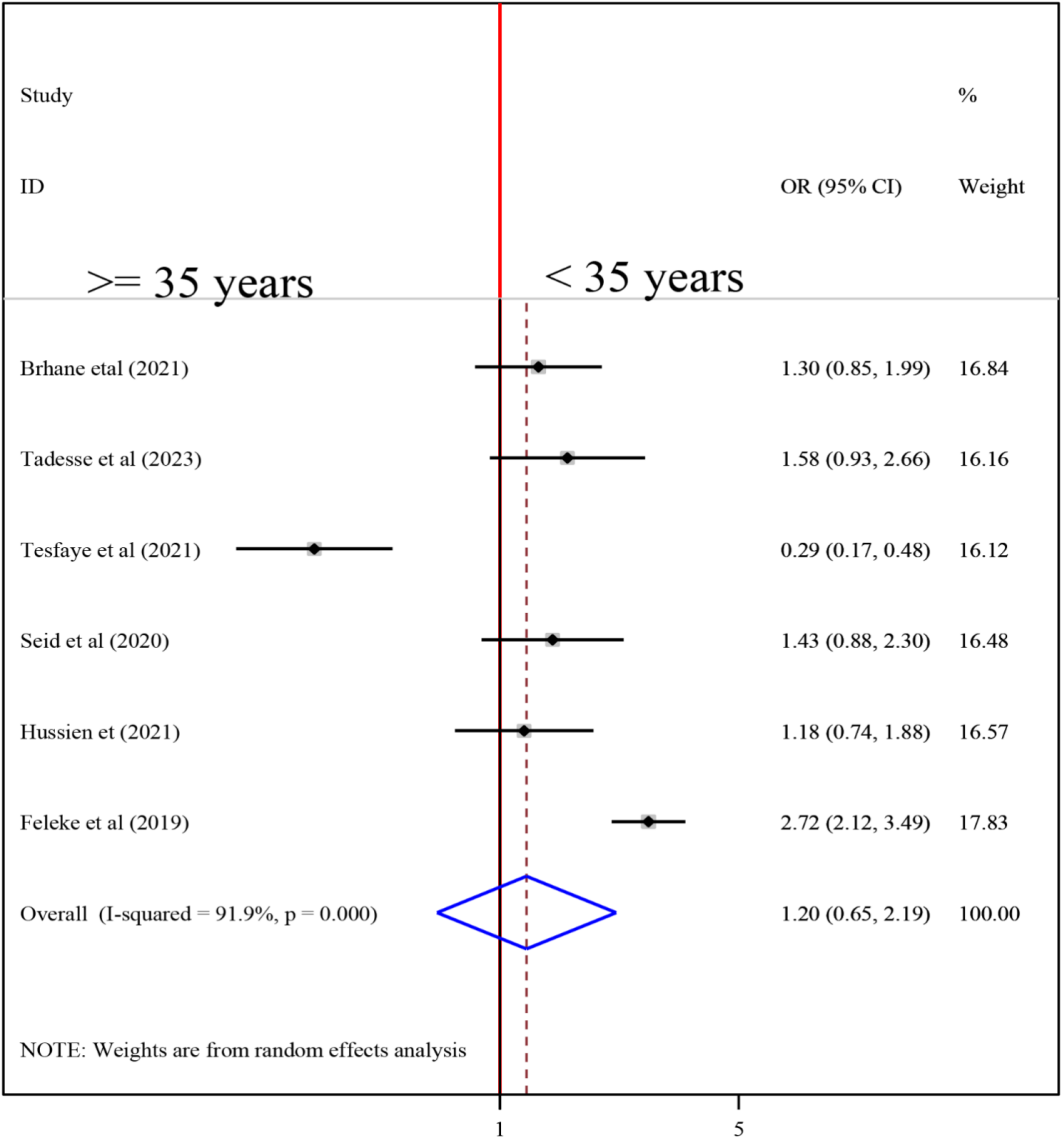
Forest plot of the pooled estimate of the association between age and undernutrition among tuberculosis patients in Ethiopia, 2023

To test the association between age and undernutrition among tuberculosis patients, six studies were included in the analysis [16, 18, 20, 21, 23, 29]. The pooled association between age and undernutrition among tuberculosis patients was assessed using a random-effects model (I2 = 91.9%, P-value < 0.001). The result of the analysis indicated that the association between age and undernutrition among tuberculosis patients was statistically insignificant (OR = 1.20 (95%CI: 0.65, 2.19) (Fig. 8).

### Association between alcohol drinking and undernutrition among tuberculosis patients

**Fig. 9:**
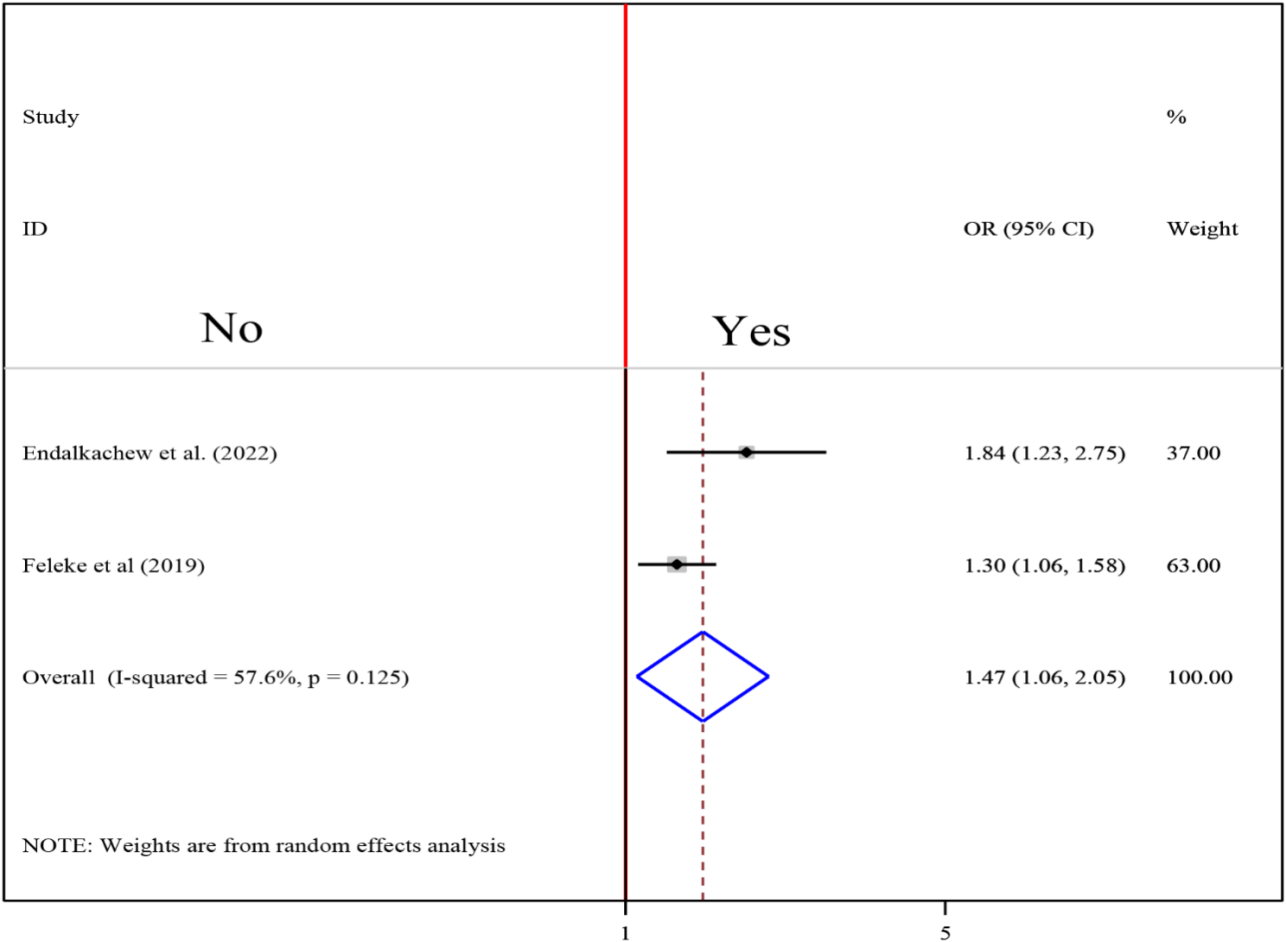
Forest plot of the pooled estimate of the association between alcohol drinking and undernutrition among tuberculosis patients in Ethiopia, 2023

To investigate the association between alcohol drinking and undernutrition among tuberculosis patients, two studies were included in the analysis [17, 29]. A random-effects model was utilized to estimate the pooled association between alcohol drinking and undernutrition among tuberculosis patients (I2 = 57.6%). The result of the analysis indicated that the association between alcohol and undernutrition among tuberculosis patients was statistically significant (OR = 1.47(95CI: 1.06, 2.05). patients who drank alcohol were 1.47 times more likely to develop undernutrition than those who did not drink alcohol (Fig. 9).

### Association between family size and undernutrition among tuberculosis patients

**Fig. 10:**
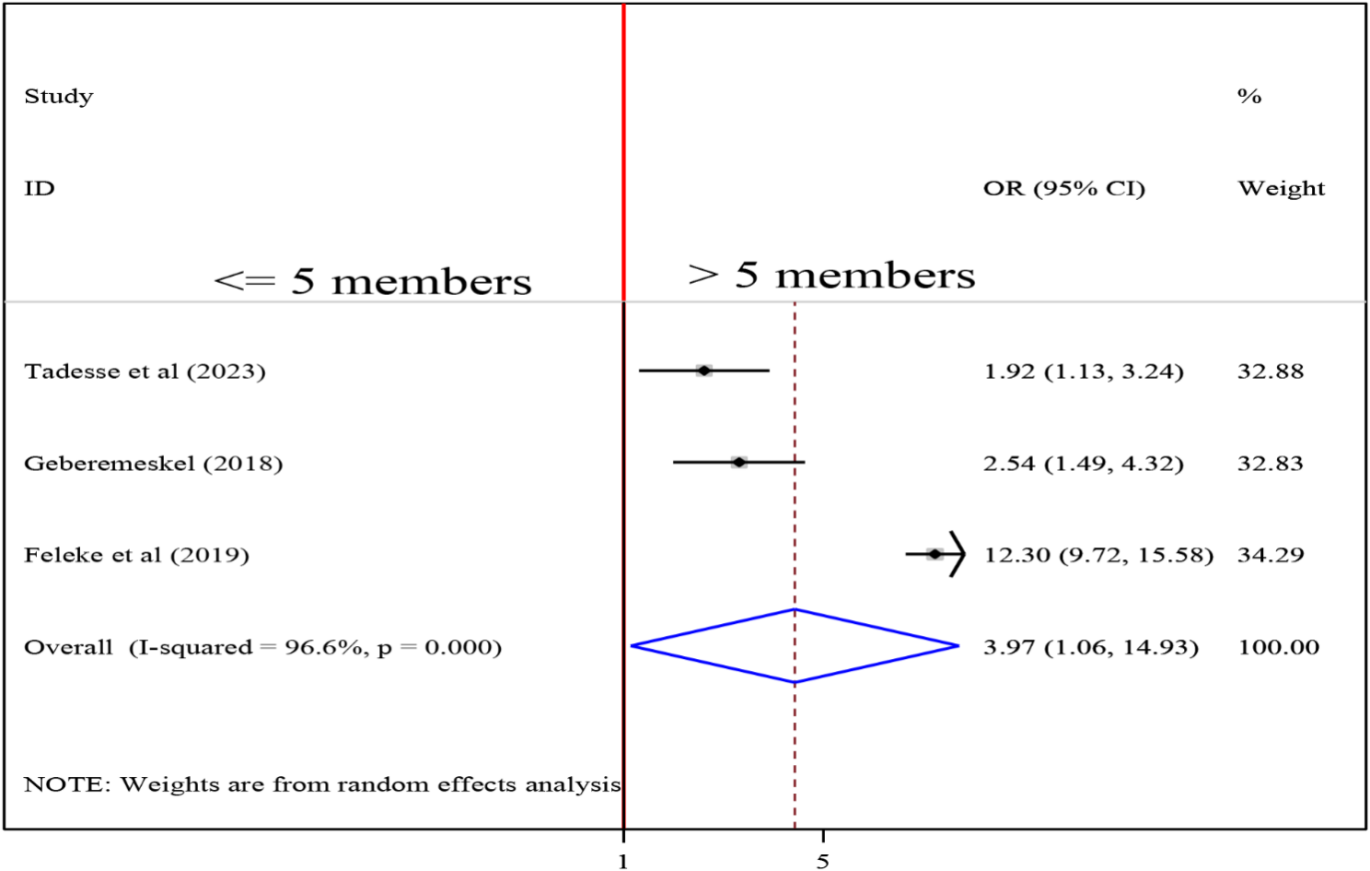
Forest plot of the pooled estimate of the association between family size and undernutrition among tuberculosis patients in Ethiopia, 2023

To detect the association between family size and undernutrition among tuberculosis patients, three studies were included in the analysis [16, 29, 30]. The pooled association between family size and undernutrition among tuberculosis patients was examined by random-effects model (I2 = 96.6%, p-value < 0.001). The pooled result of the analysis indicated that the association between family size and undernutrition among tuberculosis patients was statistically significant (OR = 3.79 (95CI: 1.06, 14.93). patients of > 5 family size were 3.97 times more likely to develop undernutrition than those whose family size < = 5 (Fig. 10).

### Association between income and undernutrition among tuberculosis patients

**Fig. 11:**
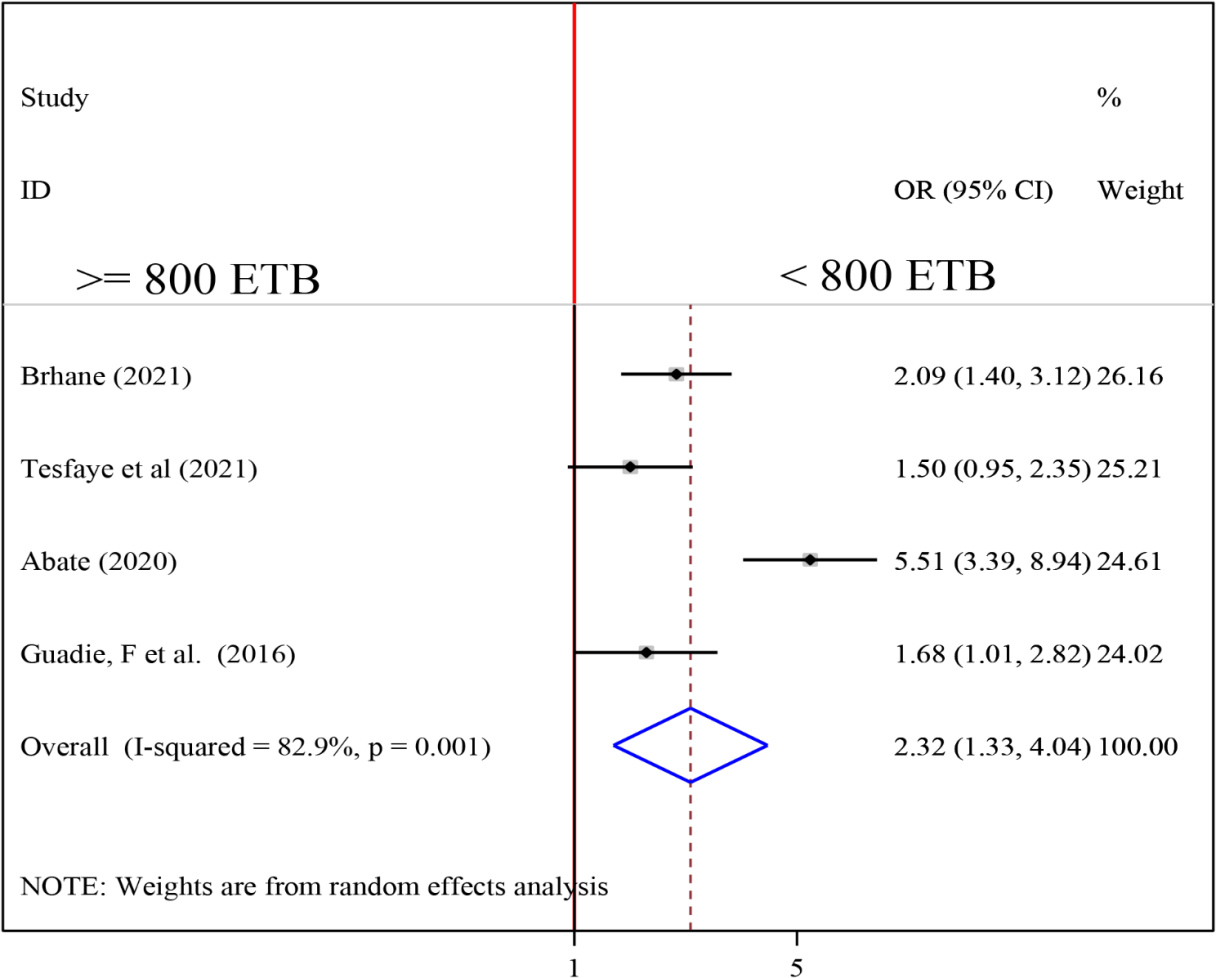
Forest plot of the pooled estimate of the association between average monthly income and undernutrition among tuberculosis patients in Ethiopia, 2023

To investigate the association between income and undernutrition among tuberculosis patients, four studies were included in the analysis [18, 21, 25, 32]. A random-effects model was utilized to determine the pooled association between income and undernutrition among tuberculosis patients (I2 = 87.6%, p-value < 0.001). The pooled result of the analysis indicated that the association between average monthly income and undernutrition among tuberculosis patients was statistically significant (OR = 2.32 (95CI: 1.33, 4.04). patients who got < 800 ETB average monthly income were 2.32 times more likely to develop undernutrition than their counterparts (Fig. 11).

### Association between functional status and undernutrition among tuberculosis patients

**Fig. 12:**
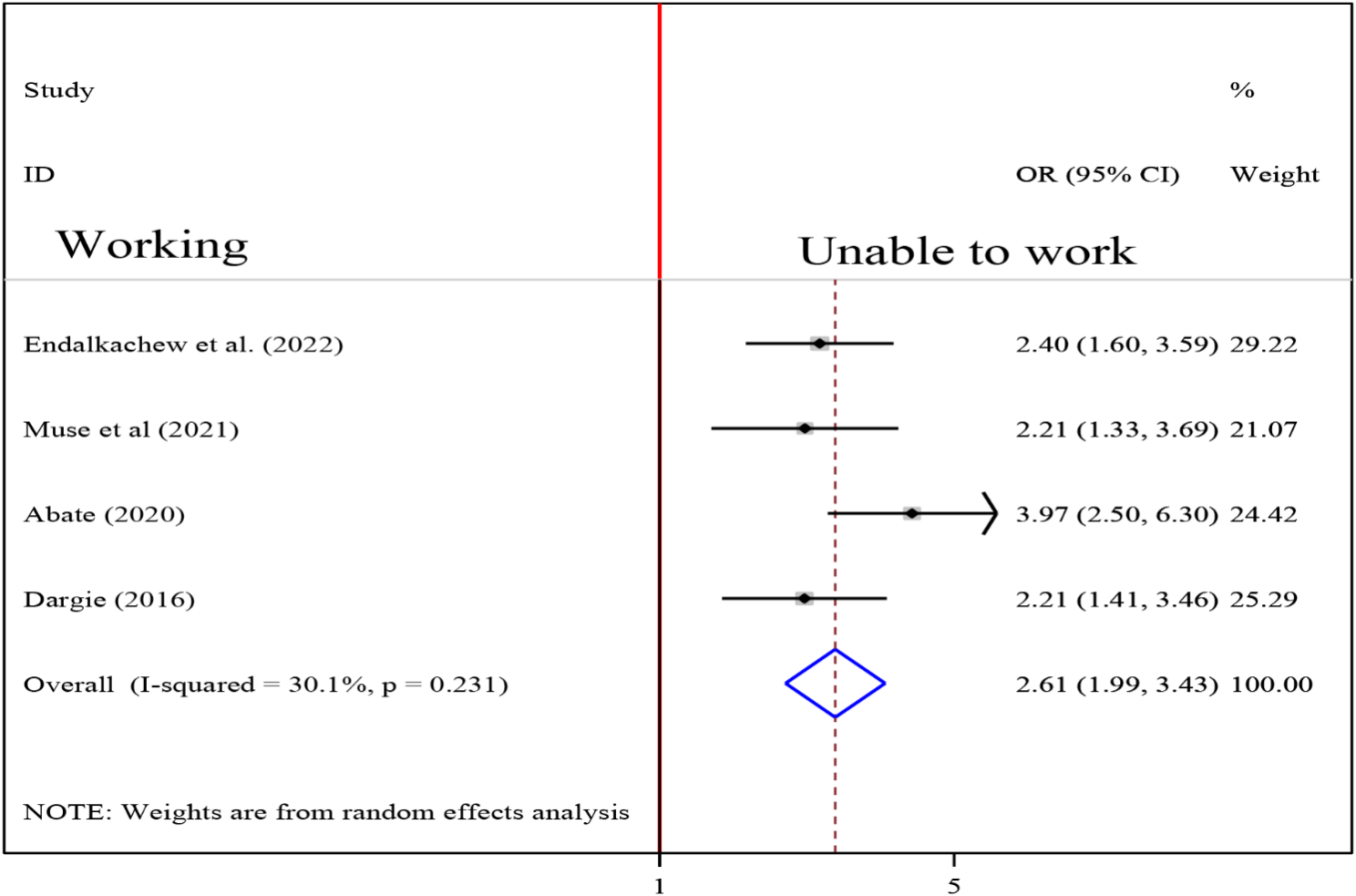
Forest plot of the pooled estimate of the association between functional status and undernutrition among tuberculosis patients in Ethiopia, 2023

To detect the association between functional status and undernutrition among tuberculosis patients, four studies were included in the analysis [17, 19, 25, 32]. The pooled association between functional status and undernutrition among tuberculosis patients was detected using fixed effect model (I2 9.9, p-value = 0.344). The analysis result indicated that the association between functional status and undernutrition among tuberculosis patients was statistically significant (OR = 2.61 (95CI: 1.99, 3.43). patients who were unable to work were 2.61 times more likely to develop undernutrition than their counterparts (fig. 12).

### Association between eating disorder and undernutrition among tuberculosis patients

**Fig. 13:**
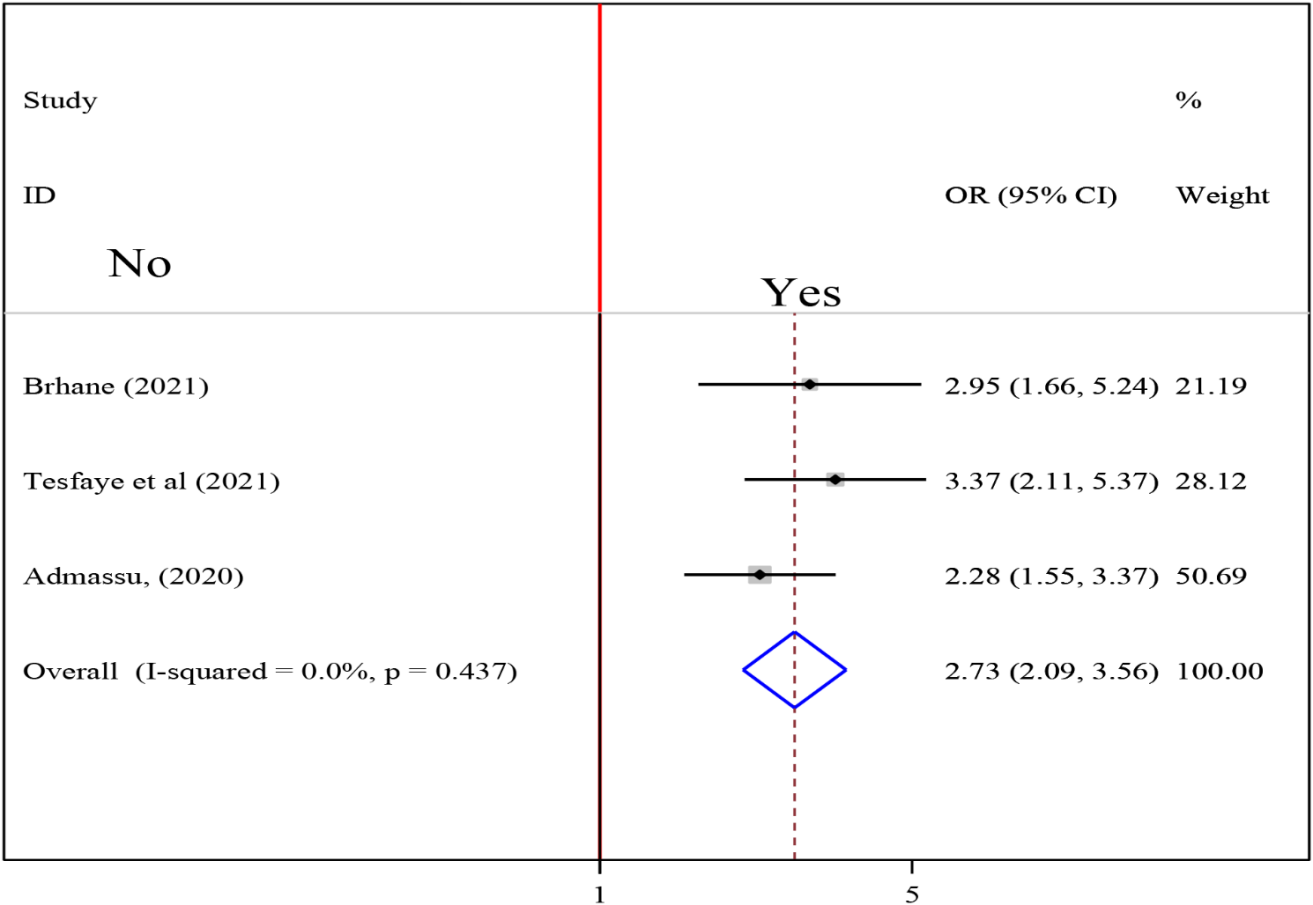
Forest plot of the pooled estimate of the association between eating disorder and undernutrition among tuberculosis patients in Ethiopia, 2023

To test the association between eating disorder and undernutrition among tuberculosis patients, three studies were included in the analysis [18, 21, 24]. The pooled association between eating disorder and undernutrition among tuberculosis patients was examined using fixed effect model (I2 0.0%, p-value = 0.437). The pooled result of the analysis indicated that the association between eating disorder and undernutrition among tuberculosis patients was statistically significant (OR = 2.73 (95CI: 2.09, 3.56). patients who had eating disorder were 2.73 times more likely to develop undernutrition than their counterparts (Fig. 13).

### Association between intestinal parasite and undernutrition among tuberculosis patients

**Fig. 14:**
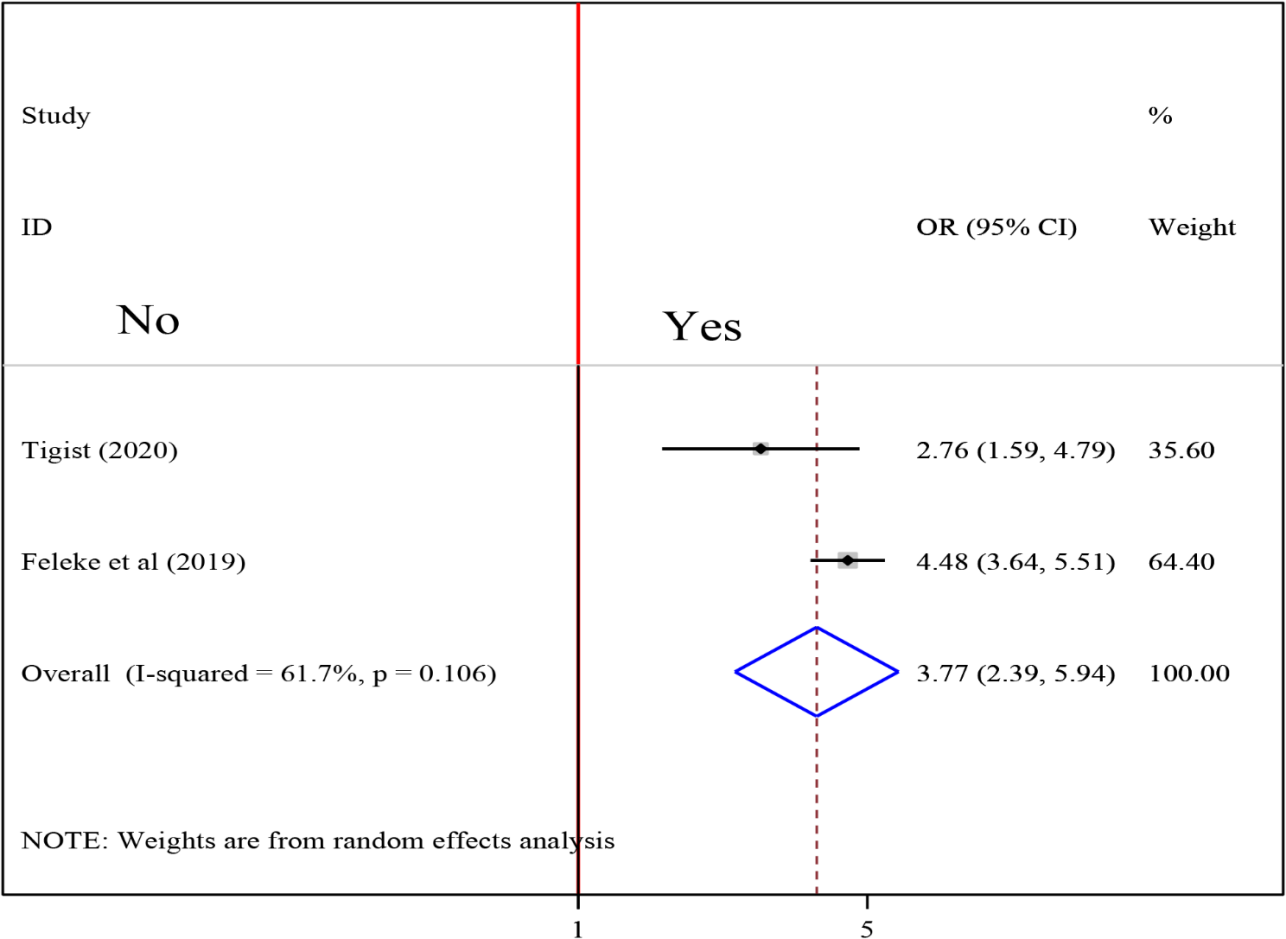
Forest plot of the pooled estimate of the association between IP and undernutrition among tuberculosis patients in Ethiopia, 2023

**Fig. 15:**
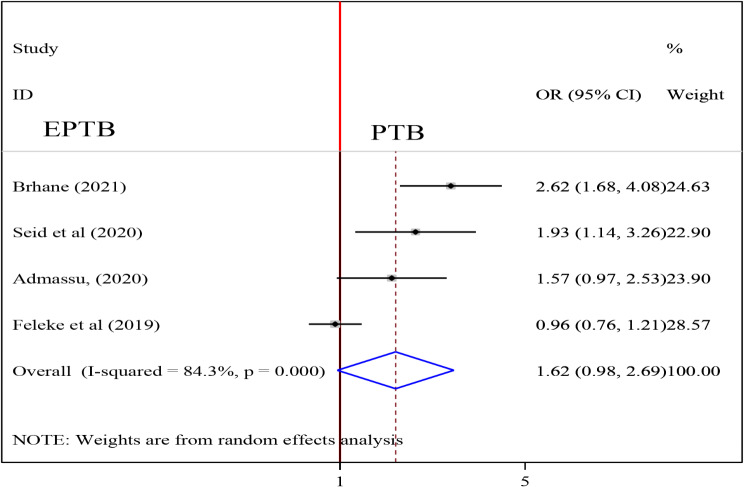
Forest plot of the pooled estimate of the association between type of TB and undernutrition among tuberculosis patients in Ethiopia, 2023

**Fig. 16:**
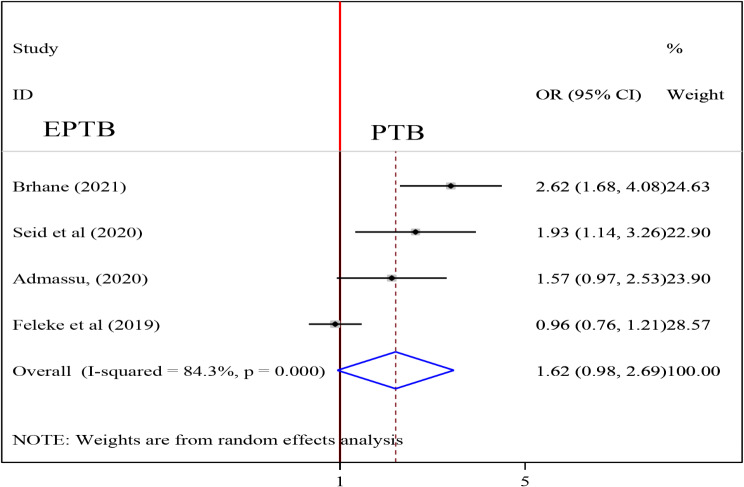
Forest plot of the pooled estimate of the association between sex and undernutrition among tuberculosis patients in Ethiopia, 2023

**Fig. 17:**
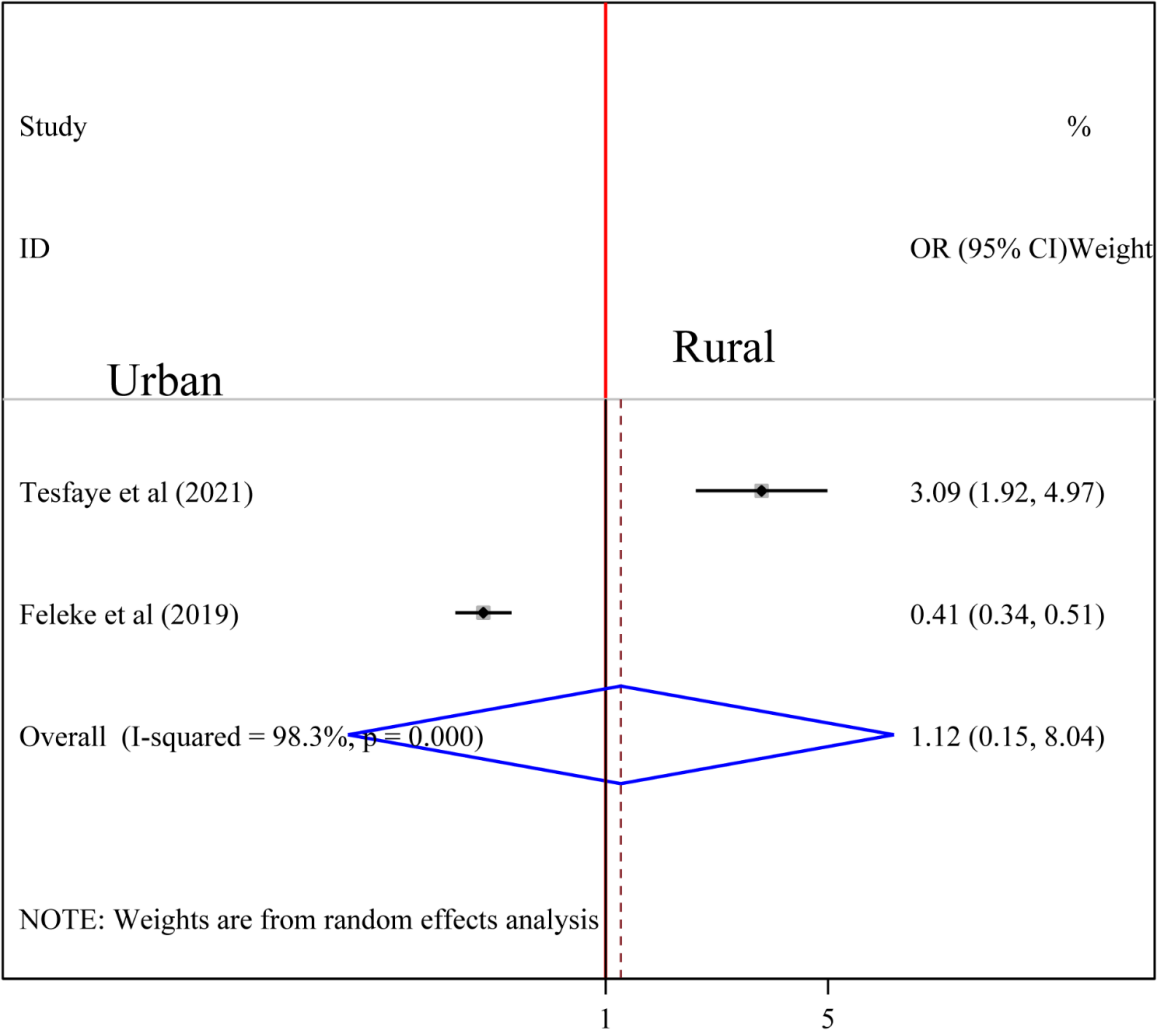
Forest plot of the pooled estimate of the association between residency and undernutrition among tuberculosis patients in Ethiopia, 2023

**Fig. 18:**
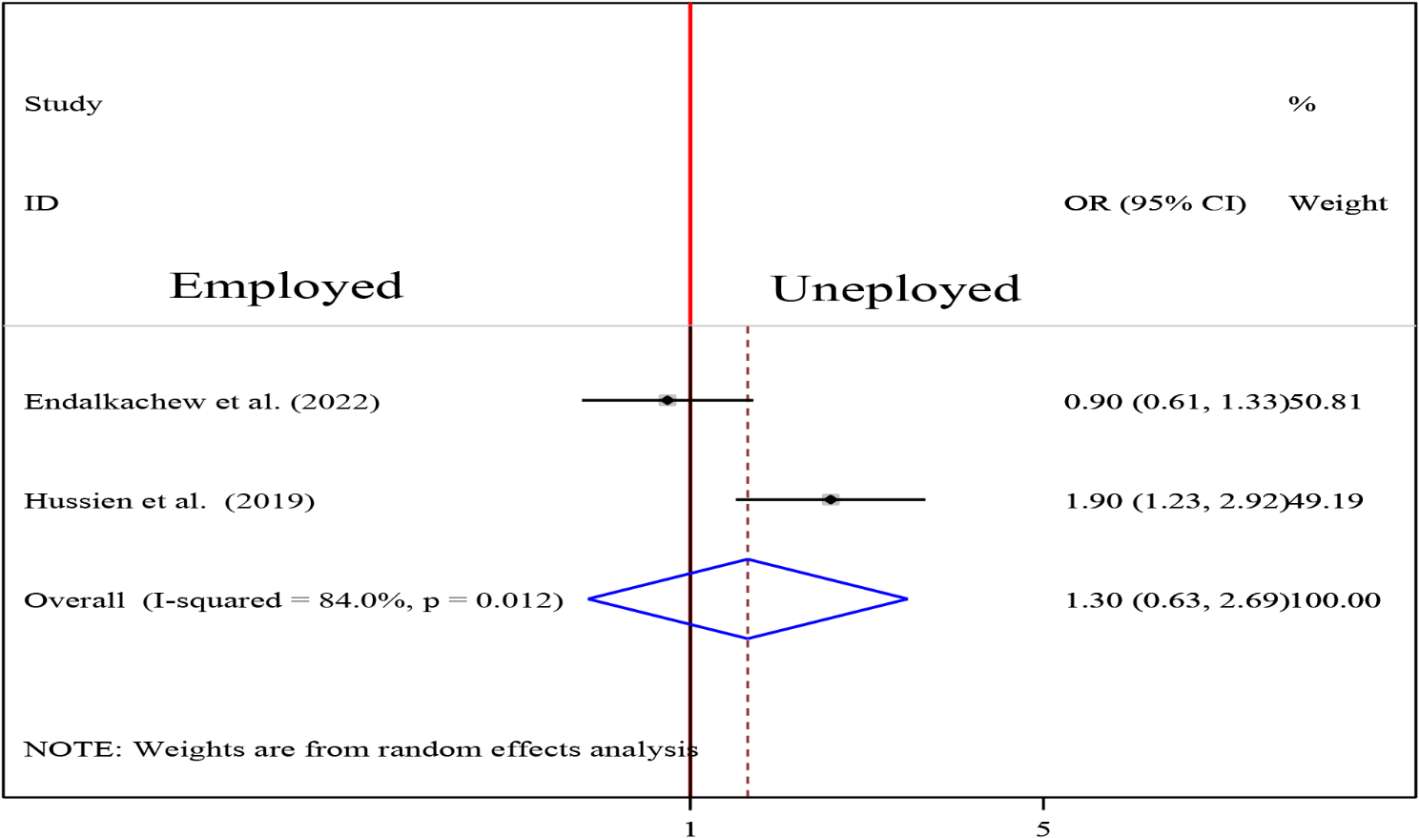
Forest plot of the pooled estimate of the association between occupation and undernutrition among tuberculosis patients in Ethiopia, 2023

**Fig. 19:**
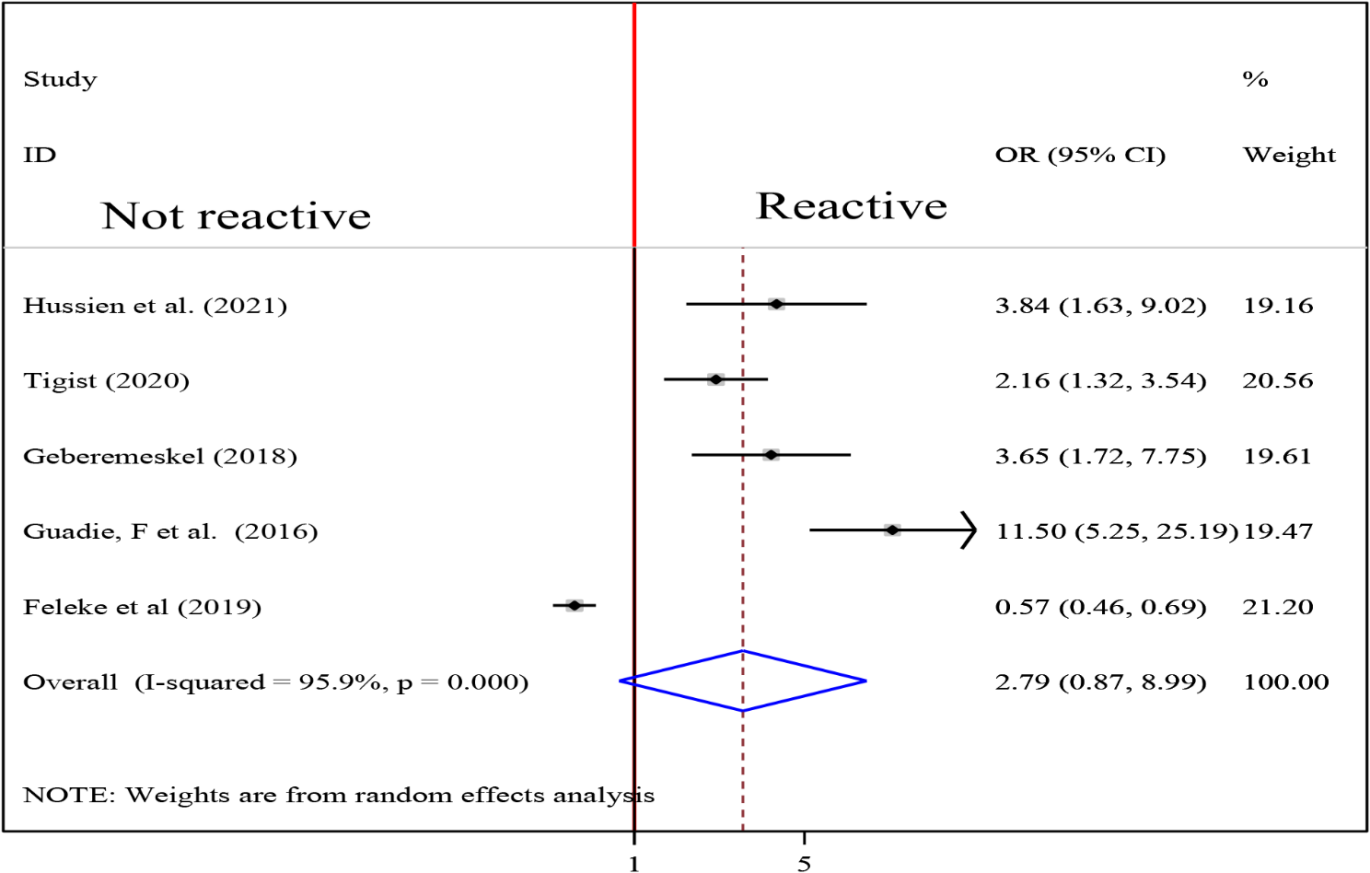
Forest plot of the pooled estimate of the association between HIV status and undernutrition among tuberculosis patients in Ethiopia, 2023

**Fig. 20:**
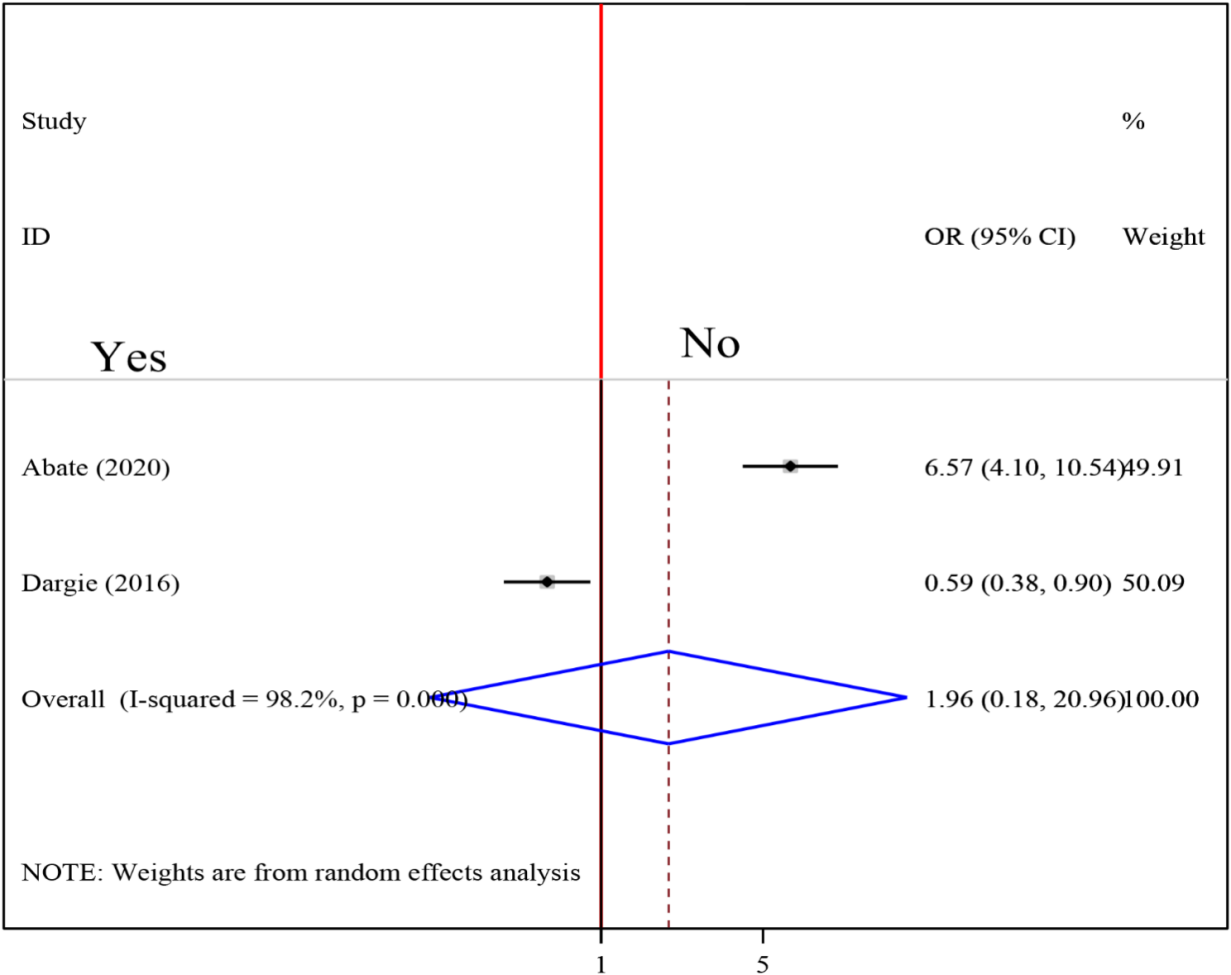
Forest plot of the pooled estimate of the association between dietary counseling and undernutrition among tuberculosis patients in Ethiopia, 2023

To examine the association between intestinal parasite (IP) and undernutrition among tuberculosis patients, two studies were included in the analysis [29, 37]. A random effects model was used to estimate the pooled association between IP and undernutrition among tuberculosis patients (I2 61.7%, p-value = 0.106). The pooled result of the analysis indicated that the association between IP and undernutrition among tuberculosis patients was statistically significant (OR = 3.77 (95CI: 2.39, 5.94). patients who had IP were 3.77 times more likely to develop undernutrition than their counterparts (Fig. 14) while the pooled results of other independent variables were statistically insignificant [Figs. 15, 16, 17, 18, 19 and 20].

## Discussion

Both undernourishment and TB are serious health issues that affect people in middle- and low-income nations. This systematic review and meta-analysis, to the best of our knowledge, is the first of its type to assess the combined magnitude and associated factors of undernutrition among patients with tuberculosis in Ethiopia. To enhance the nutritional condition of patients, which will boost the effectiveness of TB therapy and reduce TB patient morbidity and death, it is imperative to estimate the total magnitude and associated factors of undernutrition among TB patients. The results of this meta-analysis revealed that 48.23% (95% CI 42.84, 53.62) of TB patients in Ethiopia were malnourished. The finding of this study is consistent with the study conducted in Kenya (50.15%) [38], Ghana (51%) [39] and Nepal (50%) [40]. The proportional socioeconomic status of the two countries may be the cause of this parallelism.

The result of this review is higher than those of studies carried out in Bangladesh (36%) and the United States of America (11.2%) [41, 42]. This variation may be explained by disparities in the socioeconomic standing of the two countries. Ethiopia is classified as a low-income nation, the United States as a high-income nation, and Bangladesh as a middle-income nation. Additionally, this distinction could result from changes in the study.

TB patients who had no formal education were 2.11 times more likely to develop undernutrition than those who had formal education. This is because education allows people to better read and understand nutritional issues [43]. Furthermore, education provides nutritional guidance and support for tuberculosis patients [44]. The finding is supported by a study conducted in Ghana, which found that TB patients with basic and secondary education were more likely to have normal nutritional status than those who did not attend school [45]. A low maternal educational level was found to be significantly associated with a lower prevalence of overnutrition in a Colombian study (overweight or obesity). The prevalence of wasting, stunting, and anemia was higher in the lowest maternal educational categories [46]. Another study conducted in Sekondi, Ghana, found a link between educational status and malnutrition [47]. Other studies in Bangladesh found a link between education level and nutritional status, indicating that children whose mothers have a secondary or higher education have a lower risk of childhood stunting, underweight, and wasting than children whose mothers do not go to school [43].

Patients of > 5 family size were 3.97 times more likely to develop undernutrition than those whose family size < = 5. A study in Ghana also found a link between nutritional status and immediate family size [47]. Furthermore, an Indonesian study found a negative correlation between family size and undernutrition. Leaving a large and extended family was found to be associated with undernutrition in adult TB patients [48]. Another study conducted in India found that family size was significantly related to undernutrition [49]. Possible explanations for these associations include the fact that increased family size may have a negative impact on the nutritional status of every member of the household, including preschool children, because it is associated with lower per capita human inputs. Acceptance of lower quality and quantity models of fertility decisions is also implied by increased household size.

The pooled result of the analysis indicated that the association between alcohol and undernutrition among tuberculosis patients was statistically significant (OR = 1.47(95CI: 1.06, 2.05). patients who drank alcohol were 1.47 times more likely to develop undernutrition than those who did not drink alcohol. This finding is similar to that of the Ghana study, which discovered significant associations between normal nutritional status and alcohol intake status. Patients with tuberculosis who had previously or currently used alcohol were less likely to have normal nutritional status than those who had never used alcohol [45]. Many alcoholics are malnourished, either because they consume insufficient amounts of certain essential nutrients (for example, carbohydrates, proteins, and vitamins) or because alcohol and its metabolism prevent the body from properly absorbing, digesting, and utilizing those nutrients. As a result, drunkards frequently suffer from nutrient deficiencies. Again, irregular feeding habits have been linked to heavy alcohol consumption, and it has been shown that irregular feeding habits in alcoholics lead to malnutrition [50, 51].

The odds of being undernourished was about 2.3 times more likely for TB patients who got low average monthly income (< 800 ETB) than their counterparts. This finding is consistent with another study in Sri Lanka [52]. Although not a significant factor in this review, being employed was found to be a guarantee to access nutritious food in the study of Ghana [53]. The observed association could be attributed to the fact that TB diseases by itself are the disease which mainly affects poor people because of their low standard of living [54]. Those people with low income could not afford food and this might contribute for food insecurity, reduced intake, and deficiency of nutrients.

Functionality status was another factor that influenced the likelihood of undernutrition in TB patients. When compared to those TB patients who were able to work, patients who were unable to work were 2.61 times more likely to be undernourished. The possible reason for this observed association might be that a person who is unable to work has a low chance of farming, being productive and generating income which might in turn reduce the probability of getting and/or affording a variety of foodstuffs rich with nutrients. Besides, those patients who are unable to function even might be bedridden and have reduced appetite which would end with the undernutrition consequence. It was observed in the literature that appetite loss and generalized weakness that could affect working ability might lead to low dietary intake in TB patients [55]. In another way, the inability to work by itself could result from the deficiency of nutrients in the body and might be an explanation for the observed significant association between undernutrition and functionality status. In this regard, Gupta et al. pointed out that macronutrient supplementation improves functionality status in TB patients [56].

The pooled odds ratio of the included studies in this review shows that eating disorder is the significant factor for undernutrition among TB patients. Accordingly, TB patients with eating disorder had more chance of being undernourished as compared to those without eating disorder. The eating disorder could be due to loss of appetite which is one of the typical symptoms of TB disease [57]. This association can be explained by the fact that nutrient absorption is preceded by the intake and digestion of foods and any disorder to this biological process might end up with the problem of undernutrition. Patients with eating disorder might not intake, and/or digest the foods and this will contribute to inadequacy of available nutrients in the body. Because inadequate dietary intake and infections are the immediate causes of undernutrition and the association between undernutrition and TB disease is bidirectional [58, 59].

Presence of intestinal parasites is another important factor that showed significant association with the undernutrition among TB patients in this systematic review and meta-analysis. Infection with intestinal parasites was found to have 3.77 times more odds of undernutrition in TB affected patients. The study in Ethiopia confirmed that there is co-occurrence of IP and TB [60]. Other studies reported similar result that infection with intestinal parasites can affect the absorption of both macronutrients such as proteins and fats and micronutrients like zinc and Iron [61]. IP might also reduce the food intake as they are manifested with gastrointestinal disturbances including nausea [62]. These might be among the mechanisms by which IP contributes to the development of undernutrition among TB patients.

### Strength and limitation

This systematic review and meta-analysis has strengths; various databases were used to search for literature, both published and unpublished studies were searched and relevant studies were included after intensive quality assessment. Because 21 studies were included in the final analysis, it is appropriate for the exact estimation of publication bias from the funnel plot.

## Conclusion

This systematic review and meta-analysis examined the substantial magnitude of undernutrition among adult TB patients in Ethiopia. This information is crucial for understanding the scale of the problem and identifying priority areas for intervention. By knowing the prevalence rates, policymakers can allocate resources and design programs to address the specific needs of the affected population. Undernutrition was associated with a low average monthly income, Eating disorder, inability to work, having no formal education, intestinal parasite, having family size > 5 and alcohol drinking. Furthermore, a discrepancy was also found in different regions of the country. So, policy-based interventions to track these contributing factors are mandatory. The findings of this meta-analysis can guide policymakers and program developers in understanding the underlying causes and vulnerabilities related to undernutrition in TB patients.

## Data Availability

The datasets used and/or analyzed during the current study are available from the corresponding author on reasonable request.

## Abbreviations

CI: Confidence interval
OR: Odd ratio
SNNPR: Southern Nation nationality and people region
IP: Intestinal parasite
TB: Tuberculosis
